# Mechanical Characterization of 3D-Printed Patterned Membranes for Cardiac Tissue Engineering: An Experimental and Numerical Study

**DOI:** 10.3390/biomedicines11030963

**Published:** 2023-03-21

**Authors:** Aurelia Poerio, Bertrand Guibert, Mélanie M. Leroux, João F. Mano, Franck Cleymand, Jean-Philippe Jehl

**Affiliations:** 1 Institut Jean Lamour, UMR 7198 CNRS, Université de Lorraine, 54011 Nancy, France; 2 Department of Chemistry, CICECO—Aveiro Institute of Materials, University of Aveiro, 3810-193 Aveiro, Portugal

**Keywords:** biomaterials, cardiac membrane, tissue engineering, 3D bioprinting, mechanical behavior, numerical simulation

## Abstract

A myocardial infarction can cause irreversible damage to the heart muscle. A promising approach for the treatment of myocardial infarction and prevention of severe complications is the application of cardiac patches or epicardial restraint devices. The challenge for the fabrication of cardiac patches is the replication of the fibrillar structure of the myocardium, in particular its anisotropy and local elasticity. In this study, we developed a chitosan–gelatin–guar gum-based biomaterial ink that was fabricated using 3D printing to create patterned anisotropic membranes. The experimental results were then used to develop a numerical model able to predict the elastic properties of additional geometries with tunable elasticity that could easily match the mechanical properties of the heart tissue (particularly the myocardium).

## 1. Introduction

Cardiovascular diseases (CDs), notably myocardial infarction (MI), are major contributors to death worldwide [1]. When a MI occurs, the blood supply to a region of the myocardium is interrupted. In that region, the contractile cells of the cardiac muscle, the cardiomyocytes, are damaged, resulting in the necrosis of that part of the cardiac tissue with subsequent loss of the contractile capacity. This phenomenon, in turn, leads to the activation of compensatory mechanisms known as “pathological ventricular remodeling”, which can lead to heart failure [2]. For patients with end-stage heart failure, the only available treatment is heart transplantation. However, due to limitations in the number of donors, new strategies are being investigated in order to improve the cardiac function after MI and to limit (or even prevent) pathological remodeling. Among these strategies, cardiac tissue engineering, including 3D bioprinting [3], is one of the most promising. Cardiac tissue engineering strategies mainly focus on the application of passive devices, made of selected biomaterials such as epicardial restraint, or active strategies, based on the delivery of cells and/or therapeutic molecules [4]. The aim of passive devices, which can either globally surround the heart or locally reinforce the infected area, is to physically limit the pathological dilatation of the ventricle and, potentially, reverse a dilatation that has already occurred [1,5]. The two most studied epicardial restraint devices that reached human clinical trials are CorCap by Acorn (made of polyester) and HeartNet by Paracor (made of nitinol) [4,5]. Despite the promising results obtained from these two devices in reducing or reversing the progression of heart failure, associated with low or no complications post-implantation, both of them were suspended during clinical trials due to inconclusive results or to the absence of improvement compared to the control groups [2]. One limitation of these devices appears to be that while isotropic biventricular constriction is effective in preventing ventricular dilation, it might also impede diastolic filling and/or negatively impact one’s heart rate [2]. When a cardiac patch is applied to the heart wall, it is subjected to strain and shear stress caused by the heart beating. Similarly, the mechanical behavior of the region of the heart (in contact with the patch) can be modified if the mechanical characteristics of the patch are higher than those of the native tissue. The patch can then rigidify the area by applying an inappropriate force to the heart, ultimately causing a restriction effect. As a consequence, the biomaterial intended to be in close contact with cardiac tissues should exhibit similar elastic properties to the underneath tissue. The reproduction of the mechanical properties of the heart comes from a deep understanding of the structure of the myocardium [4]. Myocardium has a complex structure that is made up of three layers: the outer epicardium, the middle myocardium, and the inner endocardium. The orientation of cardiac muscle fibers (or myofibers) varies across the myocardium wall in a specific manner that confers to the heart and its particular twisting mechanism during the cardiac cycle. Furthermore, it imparts anisotropic mechanical properties to the myocardium, making it stiffer in the circumferential direction than in the longitudinal direction [6]. Due to the complexity of the myocardium, it has become clear that the knowledge and ability to reproduce its mechanical properties could be the key to the development of more sophisticated devices. A major challenge for the fabrication of cardiac patches is then represented by the replication of the structural organization of myofibers and the ability to control the degree of the structural and functional anisotropy [7]. Among the different tissue engineering technologies, such as soft lithography [6], electrospinning [8], or solvent casting [9], 3D bioprinting has emerged for the fabrication of precise and complex three-dimensional models with high repeatability. Furthermore, it allows for the direct inclusion of cells and therapeutic molecules within biomaterials (forming the so-called “bio-ink” [10]) for the production of cellularized constructs in a single step [11,12]. In this study, we evaluated a naturally derived biomaterial ink made of chitosan, gelatin, and guar gum as a promising candidate material for cardiac patches. Chitosan has successfully been used in the field of cardiac tissue engineering due to its biocompatibility and biodegradability [13]. However, its poor mechanical properties prevent its use alone as a biomaterial ink for 3D printing [14]. In this study, guar gum and gelatin were added to chitosan as thickening and gelling agents in order to improve chitosan viscosity, printability, and shape fidelity after printing. We created, by 3D printing, anisotropic membranes and characterized them by uniaxial tensile tests. Numerical simulations were then used in order to reproduce the experimental data and to develop a model allowing for the prediction of the mechanical properties of additional geometries. This approach, will not only enable a drastic reduction of the number of experiments and, thus, of time and costs, but would allow predicting the behavior of more complex membranes with mechanical properties closely matching those of the cardiac muscle.

## 2. Materials and Methods

### 2.1. Preparation and Characterization of 3D-Printed Membranes

#### 2.1.1. Materials

Chitosan (degree of deacetylation: 87.6–92.5%, viscosity: 8–15 mPas) was purchased from Heppe medical chitosan (reference 23601). Gelatin (reference 42043) was purchased from Honeywell Fluka. Guar gum (reference G4129), acetic acid (reference 33209M), and lysozyme from chicken egg white (reference 629709) were purchased from Sigma Aldrich. Ethanol absolute (reference 20821) and sodium hydroxide (NaOH, reference 28244) were purchased from VWR.

#### 2.1.2. Preparation of the Biomaterial Ink

The biomaterial ink based on chitosan, gelatin, and guar gum (CH–Gel–GG) was prepared as follows. Chitosan (20 mg/mL), gelatin (35 mg/mL), and guar gum (20 mg/mL) were dissolved in a solution of acetic acid (1%, *v*/*v*) under continuous stirring for 1 h at 50 °C. The resulting ink was then filtered through a sterile 100 µm syringe filter (Cell Strainer, pluriSelect), and either loaded into sterile syringes and kept at room temperature until the 3D printing of patterned membranes (Section 2.1.4 and Section 2.2.1), or were directly poured into Petri dishes for the fabrication of full membranes, as specified below (Section 2.2.1).

#### 2.1.3. Rheological Properties of the Biomaterial Ink

The shear viscosity of the CH–Gel–GG ink was assessed by a shear sweep analysis (between 0.1 and 100 s${}^{-1}$) at 23 °C (which corresponds to the temperature used for the printing process). The test was performed with a rotational rheometer (ARES, G2, TA Instruments) equipped with a plate geometry of 25 mm diameter.

#### 2.1.4. 3D Printing and Evaluation of the Printability

A 4th generation 3D-Bioplotter from EnvisionTEC was used to perform the 3D printing processes. The syringe temperature controller was adjusted at 23 °C and a computer-aided design (CAD) software was used to design the different structures used for this study. Either 2-layer or 10-layer 10 × 10 mm${}^{2}$ square scaffolds were used to measure the printability and the degradation of the scaffolds (see below). Scaffolds were printed using a 250 µm inner diameter needle while the speed and pressure were adapted for each experiment but were approximately 18 mm/s and 1 bar, respectively. To improve the visualization of the printed scaffolds, a blue colorant was added to the inks. The printability was evaluated using the measurement of the printability (Pr) proposed by Ouyang et al. [15] and Paxton et al. [16]. Since the fabricated object resulted in regular grids and square holes, the area (A) and perimeter (L) of the pores of each construct can be measured. The following formula results in a value that can be inferior, equal, or superior to 1:

(1) $$Pr=\frac{L^{2}}{16A}$$

When Pr = 1, the ink possesses the ideal gelation state and subsequent ideal printability since it is able to create grids with perfect square holes. Pr < 1 means that the ink is too liquid and the holes have a more circular shape while Pr > 1 means that the ink is over-gelled and results in irregular squares. Images of 2- or 10-layer constructs were analyzed using Fiji image processing software [17]. Results are presented as the average ± standard deviation of 25 measurements per condition.

#### 2.1.5. Degradation Profile of 3D-Printed Constructs

In order to investigate the degradation of the bioprinted constructs, 10-layer printed square constructs were firstly gelled by immersion in the gelling solution of 80% ethanol—2% NaOH (labeled “Et-NaOH”) for 30 min and then washed three times with PBS. After the third washing step, the excess PBS was removed using tissue paper and the samples were weighed to obtain the initial weight ($W_{i}$). They were then immersed in a solution of PBS containing 10 mg/L of lysozyme and incubated at 37 °C. At each time point (once a week from week 1 to 9) samples were removed from the lysozyme solution, wiped with tissue paper, and weighed again to obtain the final weight ($W_{f}$). At each time point, the lysozyme solution was replaced with a fresh one. The percentage of degradation was determined as:

(2) $$Weight\;loss\;\left(\%\right)=\frac{W_{i}-W_{f}}{W_{i}}\cdot 100$$

The results are presented as the average ± standard deviation.

#### 2.1.6. Dimensional Changes of the 3D-Printed Constructs after Gelation

Hydrogels tend to shrink or swell when incubated in a cross-linking solution, PBS, or water. These dimensional changes have to be taken into consideration when creating a CAD model. In fact, post-production modifications of the size may require adjustments to the theoretical model if we want to achieve a final product with precise dimensions. In our case, this is particularly important for performing accurate simulations. The dimensional stability of 3D-printed membranes was evaluated after the gelation step and after the third wash in PBS. For this study, 2-layer square structures (with a 30 mm side) were printed by overlapping a 90° filament orientation with an inter-filament distance of 2 mm. The overall size (from side to side of the square structure) and the size of the filaments were measured on the membranes immediately after printing (labeled “as printed”), on the same membranes after 30 min in the Et-NaOH (labeled “30 min Et-NaOH”) as well as after 1 h in PBS (labeled “1 h PBS”). Images were analyzed using Fiji image processing software [17]. The results are presented as the average ± standard deviation of 30 to 50 measurements for each membrane on 5 independent membranes per condition.

### 2.2. Experimental Evaluation of the Mechanical Properties of the 3D-Printed Membranes

#### 2.2.1. Creation of the Internal Pattern of Membranes by 3D Printing

**Figure 1:**
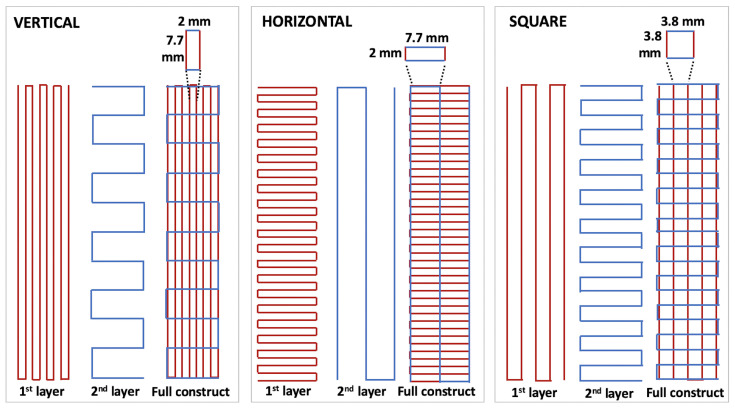
Representative images of the first two layers used for the fabrication of the 3D-printed membranes (10 layers total) with a vertical or horizontal orientation of the pores or membranes with square-shaped pores. For each orientation of the pores (vertical, horizontal, square), we show the distance between the filament used to create the geometries.

CH–Gel–GG-patterned membranes were fabricated using the previously described 3D printing process (23 °C, 250 µm conical nozzle, speed of 18 mm/s, and pressure of 1 bar). The anisotropy was created by printing 10-layer membranes with alternated (90°) vertical and horizontal filaments in order to create an internal pattern made of rectangles with a vertical or horizontal orientation with respect to the overall rectangular structure of the membrane. The distance between filaments was kept at 7.7 mm for the vertical direction and 2 mm for the horizontal direction (vertical membranes) and vice versa (horizontal membranes). Similarly, square-patterned membranes were created by printing 10 layers with alternated (90°) vertical and horizontal filaments, with a distance between filaments of 3.80 mm. The geometries of the three types of membranes (labeled vertical, horizontal, and square membranes) are schematized in Figure 1. After printing, samples were immersed in the Et-NaOH solution for 30 min, washed 3 times with PBS, and incubated overnight in PBS at 37 °C before performing the tensile tests, as specified below (Section 2.2.2). In order to investigate the evolution of the mechanical properties over time, uniaxial tensile tests were also performed on vertical and horizontal membranes kept for 7 and 14 days in PBS at 37 °C with PBS changed every other day. To prepare the full (non-patterned) membranes, the CH–Gel–GG biomaterial ink was poured into a 9 mm diameter Petri dish, treated with Et-NaOH solution for 30 min, washed 3 times with PBS, and incubated overnight in PBS at 37 °C. Rectangular sections of 8 cm × 1.6 cm were cut manually with the aid of a 3D-printed mold to perform the tensile tests.

#### 2.2.2. Uniaxial Tensile Tests

The mechanical properties of CH–Gel–GG patterned membranes were investigated via uniaxial tensile test. The test was performed using an MTS 4/ML tensile machine equipped with 100 N load cell at room temperature at a constant speed of 2 mm/min. The findings are reported through stress-strain curves and the average Young’s modulus (kPa), the average stress at break (kPa), and average elongation at break (%) ± along with the standard deviation, for multiple independent membranes under each condition (the number of membranes is indicated in the figure caption). The Young’s modulus was determined as the slope of the linear region observed in the stress–strain curve.

### 2.3. Numerical Simulation of Tensile Tests and Modeling of Additional Membranes

A finite element method was used to perform the numerical simulation of the tensile test of membranes with different internal patterns. The software COMSOL Multiphysics${}^{®}$ [18] was used both to reproduce the experimental tensile tests for (1) the determination of the Young’s modulus of the patterned experimental membranes, (2) the estimation of the Young’s modulus of the material, and (3) the prediction of the Young’s modulus of membranes with a given internal pattern.

#### 2.3.1. Geometrical Models of Membranes

The geometrical models of membranes used for the simulations of the tensile tests were built on COMSOL Multiphysics${}^{®}$ (Stockholm, Sweden) software and were based either on the dimensions of the experimentally 3D-printed membranes or on the above mentioned theoretical calculations (Section 2.1.6). Based on the purpose of the simulation, the geometry was created according to two different methods. To determine the Young’s modulus of both the membranes and the material, we reproduced the geometry of the the five membranes with vertical and horizontal orientations of the pores (labeled V1 to V5 and H1 to H5, respectively). The dimensions of the membranes (size of the filaments, thickness, width, and length of membranes) were determined experimentally and are summarized in Table S1 (Supplementary Information). For each simulation, the filament size was 0.8 mm and the thickness of the membranes was 1.85 mm (both measured experimentally). The size of the pores were calculated as percentages of the theoretical CAD design (taking into account the shrinkage of membranes).

A second method was employed to create geometries and predict the Young’s modulus of the membranes. Here, the filament size was fixed at 0.8 mm, and different combinations of pore lengths and widths (ranging from 1 to 10 mm, including 0.5 mm increments) were used to generate several membranes with specified pore sizes predicting the Young’s modulus. Here, the length always represents the size of the pore in the direction of the tensile test, while the width represents the size of the pore in the direction perpendicular to the traction. Among the different combinations was a membrane with square-shaped pores that were 2.44 mm in length and width. A tensile test was conducted to verify the model’s ability to predict the Young’s modulus of a membrane, which was then compared to the experimental results.

#### 2.3.2. Modeling of the Tensile Tests

In the model employed for the simulations, we assumed that membranes were purely elastic and made of isotropic material. To perform the simulations, we needed to determine other properties of the material, including its density, Young’s modulus, and Poisson’s ratio. We determined the Young’s modulus of the material experimentally through a uniaxial tensile test and validated it through simulations. Additionally, we experimentally measured the material’s density, which was 950 kg/m${}^{3}$. Concerning its Poisson’s ratio, we used a value of 0.45 since it falls within the range of what is expected for hydrogels [19] and is similar to that of rubber and rubber-like materials (such as several hydrogels [20]), which is of 0.5. For the simulations, we considered the membranes as fixed on the bottom and attached to a titanium bar on the top, on which the tensile force was exerted. The displacement of the titanium bar was limited to the longitudinal direction to avoid the deformation of membranes in other directions during the tensile test. The tensile test was then simulated by pulling up the titanium bar at a given force, which was too low to deform the bar. The simulation was performed either by applying the maximum force used for the calculation of the experimental Young’s modulus for vertical and horizontal membranes or fixed at 0.1 N when using the simulation to predict the Young’s modulus of membranes.

#### 2.3.3. Simulation of the Tensile Tests to Determine the Young’s Modulus of the Membranes and of the Material

The numerical simulation of the tensile test was used, first, to confirm the Young’s modulus of the five vertical and horizontal membranes, and second, to validate the Young’s modulus of the material by comparing it to the one obtained experimentally on full membranes. In order to do that, each vertical and horizontal membrane was modeled as described above (Section 2.3.1) using the dimensions of the membranes summarized in Table S1 (Supplementary Information). The simulation of the tensile test was then carried out as follows. To determine the Young’s modulus of the membranes, we applied the maximum force used during the experimental calculation of the Young’s modulus to each of the membranes. The obtained elongation was used to create the stress–strain plot (from which the Young’s modulus was calculated as the slope of the curve). The Young’s modulus of the material for this simulation was 92.3±8.6, corresponding to the one measured experimentally. Subsequently, we used the same simulations to determine the Young’s modulus of the material. By knowing the force exerted of the membranes, we determined the theoretical Young’s modulus of the material corresponding to the experimental elongation by the inverse method. The values of the force and elongation (summarized in Supplementary Information—Table S1) correspond to the last point (maximum elongation and maximum strength) of the linear region of the stress–strain curve used for the measurement of the experimental Young’s modulus.

#### 2.3.4. Statistical Analysis

The statistical significance was tested with non-parametric pairwise Wilcoxon tests with multiple testing corrections using the Benjamini–Hochberg adjustment method. A *p*-value < 0.05 was considered significant.

## 3. Results

### 3.1. Characterization of the Biomaterial Ink and the Resulting 3D-Printed Constructs

#### 3.1.1. Viscosity, Printability, and Degradation Profile

**Figure 2:**
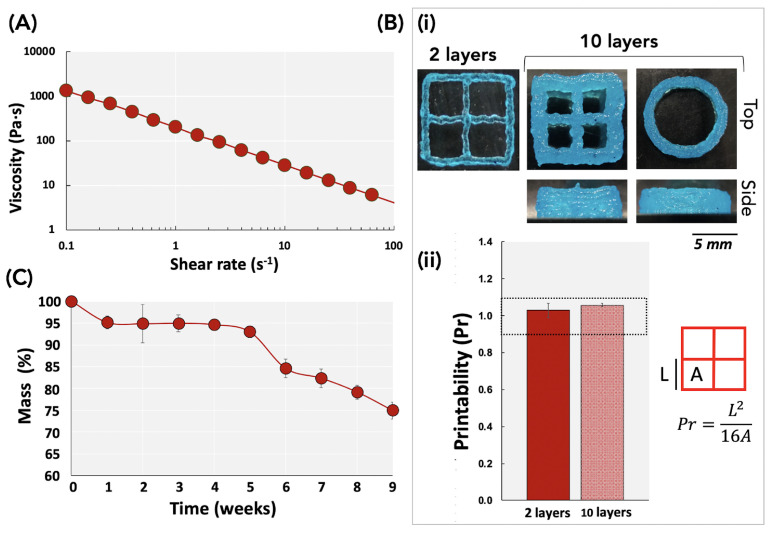
Characterization of the biomaterial ink and in vitro degradation of the 3D-printed scaffolds. (**A**) Viscosity of the ink CH–Gel–GG as a function of the shear rate, measured at 23 °C. (**B**) Evaluation of the printability of CH–Gel–GG ink. (**i**) Qualitative images of 10 mm × 10 mm square and 10 mm circular constructs printed in 2 or 10 layers are presented, viewed from the top and the side. (**ii**) Quantification of the printability (Pr value) of constructs made of 2 layers (full bars) or 10 layers (dotted bars) using the Pr value. (**C**) Degradation profile of CH–Gel–GG constructs in lysozyme at 37 °C for 9 weeks.

Viscosity: Viscosity is one of the most important parameters to consider when developing new inks or bio-inks. In fact, during 3D printing, bio-inks should be easily extruded through a nozzle under applied pressure [21]. Consequently, they should present shear-thinning behavior, meaning that viscosity decreases when the shear rate increases. Figure 2A shows the shear viscosity of the ink CH–Gel–GG as a function of the shear rate measured at 23 °C and demonstrates its shear-thinning behavior, which is suitable for 3D printing.

Printability: When new inks or bio-inks are developed, it is essential to assess their printability and shape fidelity. The latter can be defined as the ability to form 3D-printed structures with high similarity to the initial design. Here, a qualitative and semi-quantitative evaluation of the printability was conducted on 2- and 10-layer constructs made of CH–Gel–GG (Figure 2B). The qualitative evaluation of the ability of the inks to be deposed layer-by-layer, without collapsing, was done by observing the side view of the 10-layer square or cylindrical constructs (Figure 2B(i)), while the semi-quantitative evaluation of the printability (Figure 2B(ii)) was done by analyzing the shapes of the holes from the top view of the 2- and 10-layer square constructs. Qualitative images show that the ink CH–Gel–CH can be used to print at least 10-layer structures without collapsing. The representative image in Figure 2B(ii) shows the theoretical design of the constructs and evidences the area (A) and the perimeter (L) of the pores used for the measurements of the Pr value. The graph shows that the Pr values of 2-layer (full bars) and 10-layer (dotted bars) constructs printed using CH–Gel–GG ink are between 0.9 and 1.1, which represents the optimal range value to obtain good printability (see the material and method section to recall the meaning of the Pr value). Furthermore, the results show no significant difference between the Pr value measured for the 2 layers and the 10 layers, suggesting that the ink could be efficiently used for the fabrication of relevantly sized constructs.

In vitro degradation: In order to evaluate the stability of CH–Gel–GG 3D-printed constructs in a simulated in vivo environment, we studied their degradation when incubated in a solution of 10 mg/L of lysozyme at 37 °C for 9 weeks. The concentration of lysozyme was chosen to mimic the average concentration contained in the human serum [22]. The results (Figure 2C) show that, except for the initial weight loss, the degradation of the constructs was stable for at least 3 weeks while it slowly degraded during the subsequent time points (but still maintained their structure for at least 9 weeks). Based on the intended application of the constructs acting as epicardial restraints and being able to keep their shape and elasticity for a certain amount of time after implantation, this degradation profile could be appropriate. In fact, as reported in the literature, devices made of biodegradable material might be more effective than non-biodegradable materials in improving cardiac function after myocardial infarction [23].

#### 3.1.2. Dimensional Changes of the Membranes after Gelation

**Figure 3:**
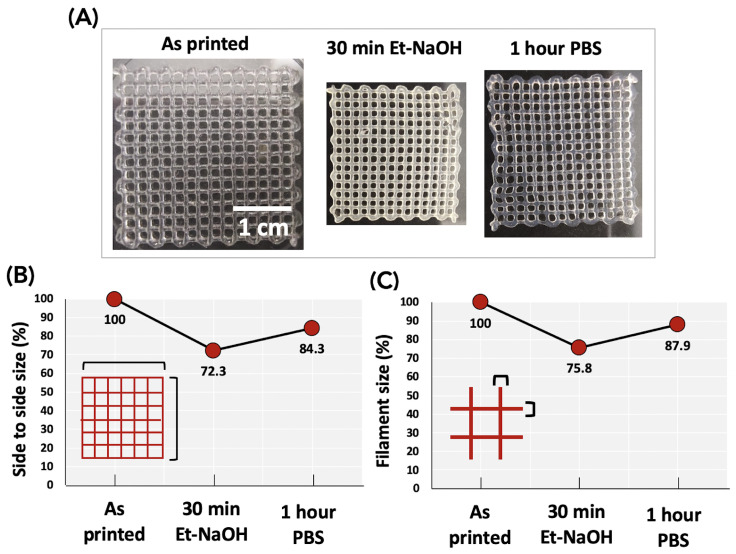
Shrinkage of 3D-bioprinted constructs after immersion in a gelling solution (ethanol and NaOH) for 30 min and subsequent swelling in PSB after 1 h. (**A**) Qualitative images of the shrinking and subsequent swelling processes. (**B**) Quantification of the variation in overall size (from side to side) of the membranes. (**C**) Quantification of the variation in filament size.

Hydrogels can show distinctive changes in their properties and physical shapes under external stimuli, such as pH, ionic strength, or temperature. Among the physical changes, hydrogels are capable of shrinking and swelling under external stimuli. These changes occur as well as during the cross-linking or gelation step, which allows hydrogels to maintain their compactness in aqueous media [24]. We noticed that CH–Gel–GG membranes would shrink after immersion for 30 min in the gelling solution (Et-NaOH) and swell when subsequently immersed in PBS. To predict the final shape of the membranes and perform the numerical simulations using the theoretical dimensions coming from the CAD model, we quantified these dimensional variations. Figure 3A shows the images of square membranes (i) as printed, (ii) after 30 min in Et-NaOH, and (iii) after immersion in PBS for 1 h. We then quantified the reduction and subsequent increment of the membrane size in two ways (Figure 3B,C). The first one (Figure 3B) is based on the measurement of the overall size of the membrane where the value of 100% is attributed to the as-printed membranes. Results show that after 30 min in Et-NaOH, the membrane sizes were reduced by 27.7% (72.3% of the initial size) while their sizes increased by 12% after immersion in PBS. Ultimately, after 1 hour in PBS, the final sizes of the membranes were 84.3% of their initial sizes. The second measurement (Figure 3C) concerns the size of the printed filaments. We found that after 30 min in Et-NaOH, the size was reduced by 24.3% (75.7% of the initial size) and was increased by 12.2% after immersion in PBS reaching 87.9% of the initial weight. We took into account the variations in the size of both the overall shape of the membranes (i.e., 84.3% of the theoretical shape) and the filament size (i.e., 87.9%) for the in silico studies.

### 3.2. Evaluation of the Mechanical Properties of CH–Gel–GG Membranes by the Tensile Test and Numerical Simulations

#### 3.2.1. Experimental Uniaxial Tensile Test

**Figure 4:**
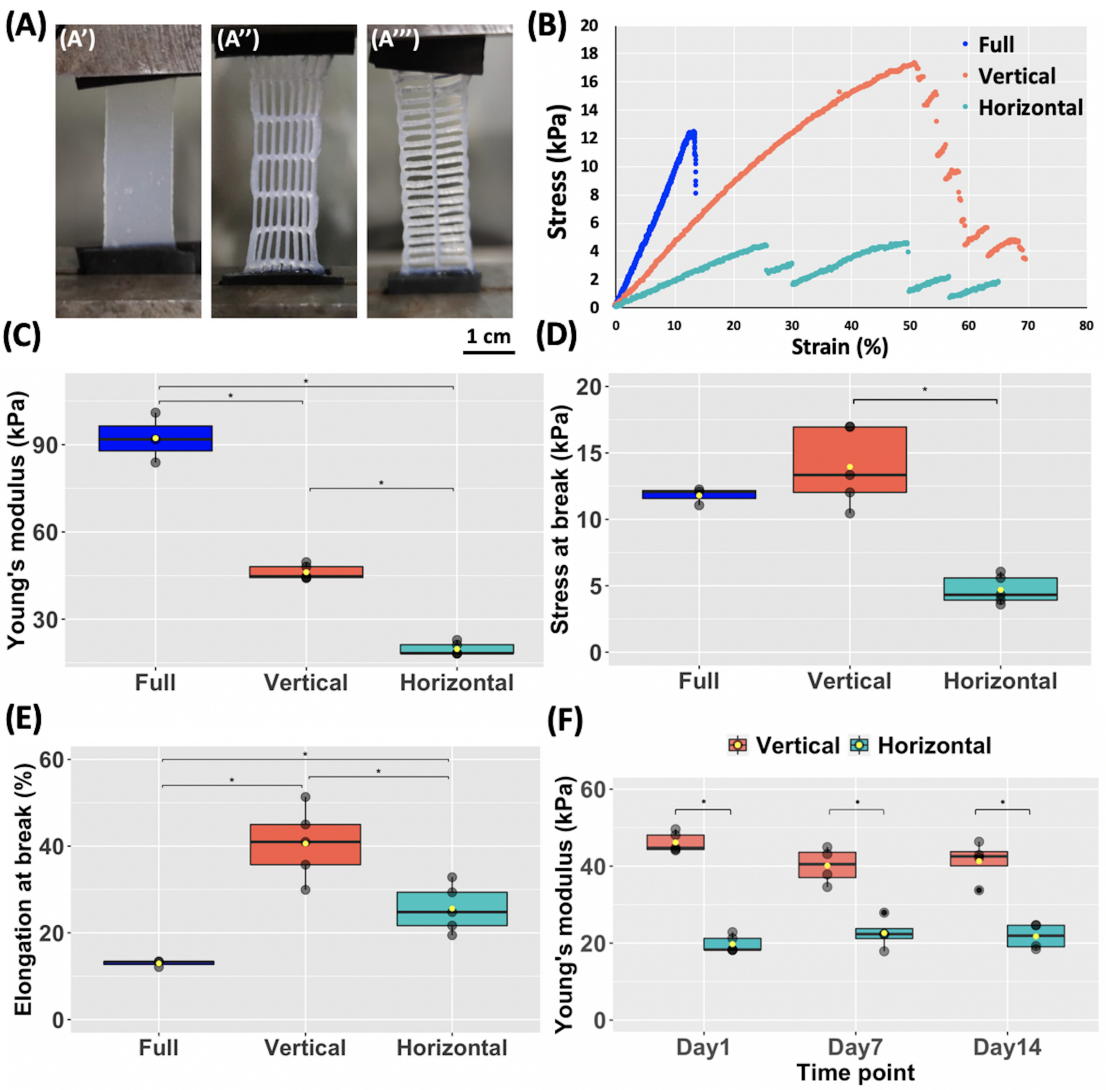
Uniaxial tensile testing of CH–Gel–GG membranes. (**A**) Photographs of (**A’**) a full membrane, (**A″**) a membrane with vertically oriented pores and (**A‴**) a membrane with horizontally oriented pores during the tensile test. (**B**) Representative uniaxial tensile stress—strain plots for full (blue), vertically (orange), and horizontally (green) pore-oriented membranes. (**C**) Average Young’s modulus calculated from the linear region of the stress–strain curve, average stress at break (**D**), and average elongation at the break (**E**) of membranes (n = 3 for full membranes and n = 5 for each group). (**F**) Average Young’s modulus of the vertically and horizontally pore-oriented membranes on day 1, day 7, and day 14 after printing and incubation in PBS at 37 °C (n = 5 for each condition on day 1, n = 4 for each condition at days 7 and 14). The boxes represent the central 50% of the data, the horizontal lines correspond to the median, and the whiskers represent the top 25% and the bottom 25% of the data. The yellow point represent the mean (* *p* < 0.05).

Mechanical properties of the printed CH–Gel–GG membranes were evaluated by the uniaxial tensile test and the obtained results are shown in Figure 4. Tensile tests were conducted on full (non-patterned) membranes (Figure 4A’), on membranes with vertically oriented pores (Figure 4A″), and on membranes with horizontally oriented pores (Figure 4A‴). The stress–strain curves (Figure 4B) show that full membranes (blue in the graph) are quite brittle and fracture at a very low elongation compared to patterned membranes, while the presence of pores improved the elastic properties of membranes. In particular, vertical membranes (orange in the graph) deform less compared to the horizontal ones (green in the graph) for the same applied stress. The linear regions of the stress–strain curves were used to calculate the Young’s modulus of membranes. As shown in Figure 4C, the full membranes have a significantly higher average Young’s modulus compared to the membranes with vertically oriented pores, which, in turn, have a significantly higher Young’s modulus compared to the membranes with horizontally oriented pores. Vertical membranes also showed significantly higher values of stress at the break (Figure 4D) and elongation at the break (Figure 4E) compared to the horizontal ones. As a consequence, the geometry of membranes could be easily modulated in order to achieve a specific Young’s modulus in a chosen direction, allowing for the fabrication of a more complex membrane. In order to assess the ability of these membranes to maintain their mechanical properties over time, we incubated vertical and horizontal membranes at 37 °C in a liquid medium (PBS) for 14 days and performed tensile tests on day 7 and day 14. Results (Figure 4F) show no significant differences in the values measured on day 1, day 7, and day 14 within the same group (vertical or horizontal) while vertical and horizontal membranes still showed significant differences among them at each time point (day 7 and day 14).

#### 3.2.2. Validation of the Young’s Modulus of the Membranes and Material

In the first step, we simulated the tensile test of the five membranes with vertically and horizontally oriented pores to reproduce the experimental results and validate them. The results of this simulation are presented in Table 1. The simulations resulted in similar values of Young’s modulus for each membrane compared to the ones measured experimentally, validating the model. In the second step, we used the same simulations to calculate the theoretical Young’s modulus of the material. In fact, since the Young’s modulus of the material is one of the most important parameters affecting the prediction of the Young’s modulus of patterned membranes, we wanted to confirm the value of the experimental Young’s modulus measured on full membranes. As a consequence, for each of the five vertical and horizontal membranes (labeled V1 to V5 for the vertical ones and H1 to H5 for the horizontal ones), we simulated a tensile test to estimate which Young’s modulus the material should have in order to be elongated at a given rate for a given applied force. The results are presented in Table 1. Keeping in mind that the Young’s modulus measured experimentally was 92.3±8.6 kPa, our results show that the simulation was able to predict (with a high accuracy) the Young’s modulus of the material for the vertical membranes (average of 92.8±2.2 kPa). However, when the Young’s modulus was calculated for the horizontal membranes, we obtained an average Young’s modulus of 98.5±13.6 kPa, which shows a higher variability. Our hypothesis is that the Young’s modulus of patterned membranes is mainly influenced by the quantity of material within the section of the membrane perpendicular to the direction of the applied tensile force. Analyzing the values in more detail, we can see that a variation of a few kPa in the Young’s modulus of membranes with a vertical orientation of pores (for example, between 44.109 kPa for V4 and 49.598 kPa for V1) results in a prediction of the Young’s modulus of the material with low variability (ranging from 91.147 kPa for V4 to 95.803 kPa for V1). The same difference in the Young’s modulus of horizontal membranes (e.g., between 18.142 kPa for H4 to 22.858 kPa for H5) results in a Young’s modulus of the material with higher variability (from 90.371 kPa for H4 to 117.710 kPa for H5). This might be due to the fact that the lower the quantity of material in the section perpendicular to the direction of the tensile test, the higher the effect of small variations in the force of traction or the elongation in the calculation of the Young’s modulus of the material itself. In fact, when the tensile tests of the membranes H2 and H5 are simulated by applying the experimental force of traction, the resulting elongation is higher compared to the experimental one, thus eliminating the variability that was calculated experimentally (values of elongation in Table S1—Supplementary Information). However, the average Young’s modulus measured with the simulation for both vertical and horizontal membranes is 95.805 kPa, which is quite similar to the one measured experimentally at 92.3 kPa, allowing us to conclude that the model could be used to validate the Young’s modulus of the material with a sufficient accuracy. These types of simulations could also be used to estimate the Young’s modulus of the material when the determination through experiments is difficult.

#### 3.2.3. Modeling the Impact of the Geometry on the Young’s Modulus of the Membrane

**Figure 5:**
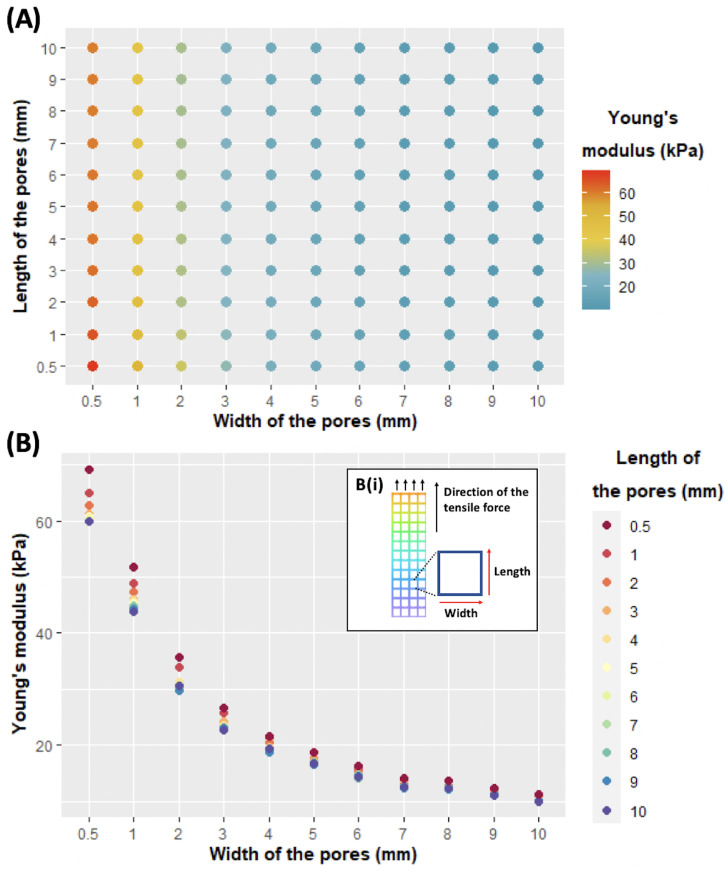
Results of the tensile test in silico of membranes with different widths and lengths of pores. (**A**) Map recapitulating the values of Young’s modulus found for each combination of the pore width and length. (**B**) Alternative representation of the effect of the width and length of internal pores on the Young’s modulus of the membranes and (**Bi**) representation of a modeled membrane clarifying the length and width with respect to the direction of the tensile test.

By knowing the Young’s modulus of the material, numerical modeling can be used to simulate tensile tests on membranes with a given geometry. Aiming to predict the elastic properties of more complex membranes with tunable local stiffness, we used this model to estimate the Young’s modulus of membranes with variable sizes of internal pores. In particular, we tuned the length and width of the pores (every mm, from 1 to 10 and including 0.5) and modeled uniaxial tensile tests in the direction of the length. On the contrary, regarding the thickness of the membrane, the Young’s modulus of the material and the size of filaments were fixed at 1.85 mm, 92.3, and 0.8 mm, respectively, which corresponded to the values measured experimentally. The overall size of the membrane (width and length of the membrane) varied according to size and the number of pores (values in Table S2—Supplementary Information). Results (Figure 5A) show that by using the same material (CH–Gel–GG in our case) and simply adjusting the length and width of the pores we can create membranes with several internal patterns with a Young’s modulus ranging between 10 and 70 kPa. The results in Figure 5B show that the Young’s modulus of the membrane increases when reducing the width and/or the length of the pores, as expected. The results also show that for small-width pores, the impact of the length on the resulting Young’s modulus is more pronounced. For example, considering a width of 10 mm, there is a difference of only a few kPa in the value of the Young’s modulus between the two extreme values of the length (being 10.1 kPa when the length is 10 mm and 11.3 kPa when the length is 0.5 mm). However, for the membrane with a pore width of 0.5 mm, the difference in the value of the Young’s modulus when the length varies from 0.5 to 10 mm is around 10 kPa (59.8 kPa for a length of 10 mm and 69.1 kPa for a length of 0.5 mm). Altogether, our results show that variations in the width of the pores have a higher effect on the elasticity of the membrane compared to the length of the pores. According to our hypothesis, this is due to the fact that when the width of the pores decreases, it means that there is a higher amount of material within the section of the membrane perpendicular to the direction of the traction.

#### 3.2.4. Validation of the Model through Experimental Confirmation

**Figure 6:**
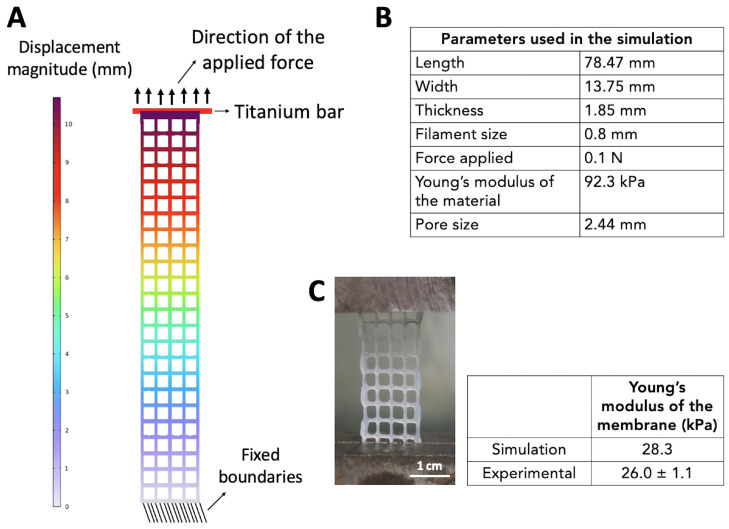
Simulation and experimental results of the tensile test of the square membranes. (**A**) Model of the square membrane after elongation under an applied force of 0.1 N. (**B**) Table summarizing the parameters used in the simulation. (**C**) Representative photograph of a square membrane during a tensile test and table summarizing the simulation and experimental results (n = 8).

Here, we assess the ability of our model to predict the Young’s modulus of a membrane with a square geometry (as represented in Figure 6A) and experimentally validate the prediction of the model. For the simulation, again, we assumed that the Young’s modulus of the material was 92.3 kPa and that the size of the filament was 0.8 mm. The length and width of the pores were chosen to be 2.44 mm and the length and width of the membranes varied according to the sizes of the pores and filaments, as summarized in Figure 6B. According to the numerical simulation, square membranes should have a Young’s modulus of 28.3 kPa when subjected to a tensile force of 0.1 N. We then 3D-printed the square membranes, performed the tensile test (Figure 6C), and obtained a Young’s modulus of 26±1.1 kPa, which is quite close to the numerical prediction.

## 4. Discussion

The aim of this study was to evaluate the feasibility of using an entirely naturally derived biomaterial for the development of cardiac patches with anisotropic behavior able to match the local mechanical characteristics of the myocardium. In fact, when a cardiac patch is applied to the heart wall, it is subjected to strain and shear stress caused by the heart beating. Similarly, the mechanical behavior of the region of the heart in contact with the patch can be modified if the mechanical characteristics of the patch are different from those of the native tissue. The patch can then rigidify the area, by applying an inappropriate force to the heart, ultimately causing a restriction effect. After an MI, the restriction of the region undergoing pathological expansion might be the aim of the application of a cardiac patch. In this regard, it appears very helpful to locally tune the mechanical properties of the patch by easily creating a personalized geometry through 3D printing. Testing (through numerical simulations) the different geometries in order to select the aimed values of elasticity for the fabrication of a more complex patch would be even more helpful. In order to do that, we developed an entirely natural biomaterial ink made of chitosan, gelatin, and guar gum, which are all known to be biocompatible components [25,26]. We showed that this biomaterial ink can efficiently be 3D-printed in a given geometry. We performed uniaxial tensile tests to evaluate the elasticity of membranes made by the solvent casting method as well and membranes with a vertical and horizontal orientation of pores made by 3D printing. While the full membranes are rigid and brittle, 3D-printed membranes show a higher resistance to traction in terms of elongation at the break. Furthermore, vertical and horizontal membranes showed values of Young’s modulus significantly different from each other, confirming that the same biomaterial can be used to easily tune the elastic properties of patterned membranes. As a consequence, aiming to develop more complex cardiac membranes with specific local elasticity, we developed a numerical model able to both validate the experimental results and predict the Young’s modulus of additional geometries. By simulating the tensile test of the patterned 3D-printed membranes (vertical and horizontal) we calculated the theoretical Young’s modulus of both the membranes and the material. The values of Young’s modulus of the membranes were very similar to the ones obtained experimentally, validating the efficiency of the model. Using the same simulations of the vertical and horizontal membranes we estimated the values of Young’s modulus of the material, which were close, on average, to the one measured experimentally. The values of Young’s modulus were more similar when the simulation reproduced the tensile test of membranes with vertically oriented pores compared to membranes with horizontally oriented pores. This variability can be explained by the fact that while a membrane modeled and used for the numerical simulation is perfect, 3D-printed membranes present some imperfections. Furthermore, stress is applied to the membranes when they are experimentally placed on the tension machine and fixed with the two clamps. In some cases, this force might weaken the membrane, resulting in slightly altered values of force and elongation. In addition, some membranes mounted on the tensile machine may not be perfectly straight, such as the modeled one, resulting in a more variable deformation. Considering that the amount of material in the section perpendicular to the direction of the traction is much lower in the horizontal membranes (made of three filaments) compared to the vertical ones (made of 8 filaments), it is not surprising that small alterations have a stronger effect on the calculation of the Young’s modulus of the material. Since the average value of Young’s modulus of the material obtained from the simulation was similar to the one measured experimentally, we used the latter to model tensile tests of additional geometries with internal pores of different sizes. By varying the width and length of the pores, we were able to cover a pretty large range of values of Young’s modulus, from 10 to 70 kPa. The values of Young’s modulus of the myocardium reported in the literature vary according to (a) the species considered, (b) the moment of the cardiac cycle at which the Young’s modulus is measured (i.e., systole or diastole), and (c) the technique used to measure it. For example, Engelmayr et al. [27] used uniaxial tensile tests to evaluate the elastic modulus of circumferentially or longitudinally oriented rectangular specimens obtained from the right and left ventricular myocardium of adult rats. Their results showed that the right ventricular myocardium had a stiffness (E) of 54±8 kPa in the circumferential direction and 20±4 kPa in the longitudinal one, while the left ventricle had a stiffness of 157±14 kPa in the circumferential direction and of 84±8 kPa in the longitudinal one. The authors of this study proved the macroscopic anisotropy of the myocardium, based on the direction of the traction (circumferential or longitudinal, with respect to the apex-to-base axis). However, these mechanical properties are due to a way more complex organization of myofibers within the heart wall, which can be evaluated at a more microscopic level. In this regard, Jehl et al. [28] characterized the mechanical properties of the myocardial wall of pig cardiac tissue by performing nanoindentation measures on tissue slices of the long axis of the left ventricle. Their results showed variations in stiffness according to the local orientation of myofibers within the myocardial tissue. In particular, it was discovered that the tissue is less stiff, resulting in a Young’s modulus of 10 kPa, when the indentation is parallel to the fibers, meaning that the fibers are longitudinally oriented. Conversely, the tissue is stiffer, resulting in a Young’s modulus of 250 kPa, when the indentation is perpendicular to the fibers, meaning that the fibers are radially oriented; they were able to describe and model the overall fiber orientation. Concerning our developed cardiac membranes, the above-mentioned range of elasticity (from 10 to 70 kPa) was obtained using a material with a Young’s modulus of 92.3 kPa, a size of the filament of 0.8 mm, and a thickness of the membrane of 1.85 mm; thus, it can be modulated by modifying these parameters. As a consequence, it is clear that the mechanical properties of the membranes can be tuned by selecting a different biomaterial, by modifying some of the parameters of the membrane fabrication, or by modifying the structure (pattern). An example of how a material can be modulated to tune its mechanical properties was shown by Engelmayr et al. [27]. The authors of this study, after having evaluated the mechanical properties of rat hearts, created anisotropic membranes based on PGS (poly(glycerol sebacate)) by fabricating accordion-like honeycomb patterns. This particular design leads to higher stiffness (195 ± 8 kPa) in a direction compared to its orthogonal one (57 ± 3 kPa). However, the authors also showed that by varying the curing temperature of PGS they were able to modulate the elastic properties of the membranes and the anisotropy ratio. However, for a given set of parameters, the model we propose can be used as a guide to choose the sizes of the internal pores and predict the elasticity of the whole membrane. Furthermore, except for membranes with square-shaped pores, which have isotropic transverse behaviors, membranes with rectangular pores show different elasticities in the two directions of the rectangular pores (orthotropic behavior). As a consequence, the fibrous myocardium has radial and longitudinal elasticity, and a cardiac membrane with rectangular-shaped pores will have two distinct elastic behaviors in the two directions, potentially matching the ones of the myocardium. For example, in our study, vertical and horizontal membranes have the same size of pores but result in a Young’s modulus of 42.6 kPa when rectangular pores are aligned vertically and of 19.8 kPa if pores are aligned horizontally with respect to the direction of the traction. Similar values of elasticity were found by Engelmayr et al. [27] when performing a tensile test on the right ventricle (54±8 kPa for the circumferential direction and of 20±4 kPa for the longitudinal one). In order to validate our model, we simulated the tensile test of membranes with square-shaped pores and then performed it experimentally. Values obtained experimentally and from the simulation were close to each other (26.0±1.1 kPa for the experimental one and 28.3 kPa for the simulation) allowing us to validate the ability of the model to predict the Young’s modulus of a patterned membrane with sufficient accuracy.

## 5. Limitations of the Study

**Figure 7:**
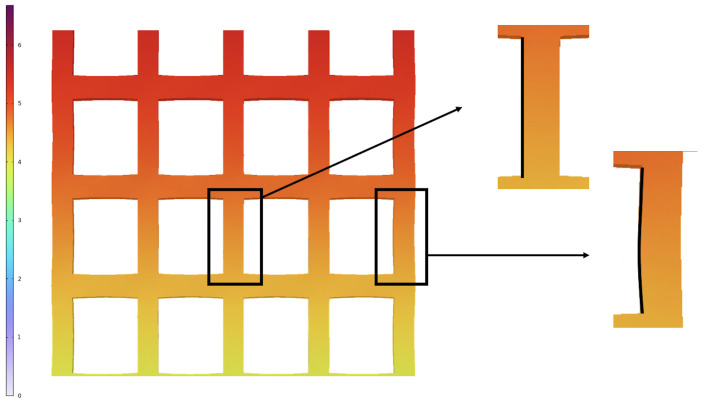
Representative image of the middle part of the square membrane during the simulation of the tensile test where deformations can be appreciated.

The Poisson’s ratio of the biomaterial used in this study was selected based on the literature (i.e., 0.45) [19]. However, further studies will be necessary to better investigate the deformation of both the membrane itself and the material around each pore, taking into account their positions within the membranes. In fact, as shown in Figure 7, the material around the pores located in the center of the membrane deforms less than the material surrounding the pores located on the side.

## 6. Conclusions and Perspectives

In this study, we characterized (by the uniaxial tensile test) the mechanical properties of chitosan–gelatin–guar gum membranes. We showed that by using 3D printing to create patterned membranes, we significantly improved the elasticity and resistance to traction of the same material. We developed a numerical model in order to (a) validate the experimental results and (b) predict the elasticity of membranes with a given internal pattern. This strategy will allow us to model complex membranes with tunable elastic properties that are able to closely match the anisotropy of the myocardium. In the next step, the ability of the membranes developed here to withstand the cyclic fatigue test will be evaluated. As a consequence, considering the heartbeat rate, it may be necessary to adjust the model to account for viscoelasticity.

## Figures and Tables

**Table 1:** Table summarizing the results of the calculation of the Young’s modulus of the membranes and of the material, both experimentally and in silico. In more detail, the table summarizes the values of Young’s modulus measured experimentally on five vertical (V1 to V5) and five horizontal (H1 to H5) membranes. The dimensions of the membranes and the values of elongation and force used To determine the experimental Young’s modulus of the membrane were employed to determine the Young’s modulus of the membranes in silico. These data were used to calculate by simulation the Young’s modulus of the material in order to validate the one measured experimentally (92.3±8.6). Values of the maximum elongation and force, as well as the dimensions of the membranes (length, width, and thickness) can be found in Table S1—Supplementary Information.

		Young’s Modulus (kPa)			
Type of		Membrane		Material	
Membrane	Sample	In Silico	Experimental	In Silico	Experimental
	1	45.2	49.6	95.8	
	2	43.9	44.7	91.5	
Vertical	3	44.2	44.3	91.2	
	4	45.0	44.1	91.1	
	5	45.9	48.0	94.8	92.3±8.6
	1	18.6	18.3	89.7	
	2	18.3	21.2	107.6	
Horizontal	3	18.9	18.3	88.3	
	4	17.4	18.1	90.4	
	5	17.5	22.9	117.7	

## Data Availability

Data supporting the reported results of the experimental studies and numerical simulations can be found in the Supplementary Materials.

## Supplementary Materials

The following supporting information can be downloaded at https://www.mdpi.com/article/10.3390/biomedicines11030963/s1, Table S1: Summary of the experimental parameters of the ten membranes used for the simulations and results of the simulation; Table S2: Summary of the dimensions of the membranes and internal pores used for the simulations of the different patterns.
