# Supplementary material for: Mechanical Characterization of 3D-Printed Patterned Membranes for Cardiac Tissue Engineering: An Experimental and Numerical Study
Source: Biomedicines. 2023 Mar 21;11(3):963. doi: 10.3390/biomedicines11030963 (PMC10046740; doi:10.3390/biomedicines11030963)
Supplement: Supplementary file 1 [file biomedicines-11-00963-s001.zip › biomedicines-2197348-supplementary.pdf]

Supplementary Material

Table S1. Summary of the experimental parameters of the ten membranes (five vertical and five horizontal) used for the simulations and results of the simulation (elongation and Young's modulus). The size of the filament was always 0.8 mm, the thickness of the membranes 1.85 mm and Young's Modulus of the material 93.267 kPa.

Type of Membrane	Sample	Length (mm)	Width (mm)	Force (N)	Elongation (mm)		Young's Modulus (kPa)	
					Experimental	In Silico	Experimental	In Silico
Vertical	1	46.87	13.61	0.236	9.385	9.728	45.154	49.598
	2	48.48	13.79	0.224	9.706	9.610	43.948	44.730
	3	52.15	14	0.227	10.459	10.352	44.196	44.344
	4	41.03	13.65	0.225	8.219	8.088	45.025	44.110
	5	48.61	13.25	0.235	9.737	10.051	45.905	48.068
Horizontal	1	44.55	13.23	0.089	8.963	8.749	18.618	18.305
	2	50.67	13.48	0.107	10.135	11.814	18.304	21.228
	3	52.93	13.02	0.087	10.619	10.046	18.850	18.269
	4	56.39	14.02	0.088	11.126	10.828	17.354	18.142
	5	51.26	14.02	0.111	9.766	12.418	17.527	22.858

Table S2. Summary of the dimensions of the membranes and internal pores used for the simulations of the different patterns (each line represent a membrane with a specific pattern). The size of the filament was always 0.8 mm, the thickness of the membranes 1.85 mm, the maximum force 0.1 N and the Young's Modulus of the material 93.267 kPa.

Pores		Membranes		Number of Filaments		Results	
Length (mm)	Width (mm)	Length (mm)	Width (mm)	Perpendicular to the Stretching	Parallel to the Stretching	Elongation (mm)	Young's Modulus (kPa)
1	1	89	17	50	10	5.684	48.952
1	2	89	17.6	50	7	8.130	33.895
1	3	89	19.8	50	6	9.596	25.735
1	4	89	20	50	5	11.533	21.308
1	5	89	18.2	50	4	14.355	18.674
1	6	89	21.2	50	4	14.485	15.889
1	7	89	24.2	50	4	14.622	13.897
1	8	89	18.4	50	3	19.485	13.636
1	9	89	20.4	50	3	19.125	12.194
1	10	89	22.4	50	3	19.745	11.040
1	0.5	89	19	50	15	3.862	64.862
2	1	82	17	30	10	5.445	47.358
2	2	82	17.6	30	7	7.943	31.368
2	3	82	19.8	30	6	9.308	24.048
2	4	82	20	30	5	10.895	20.591
2	5	82	18.2	30	4	14.076	17.586
2	6	82	21.2	30	4	13.671	15.223
2	7	82	24.2	30	4	14.165	13.137
2	8	82	18.4	30	3	18.973	12.870
2	9	82	20.4	30	3	18.423	11.664
2	10	82	22.4	30	3	18.542	10.672
2	0.5	82	19	30	15	3.702	62.856
3	1	92	17	25	10	6.246	46.037
3	2	92	17.6	25	7	9.005	31.379

3	3	92	19.8	25	6	10.413	24.314
3	4	92	20	25	5	12.835	19.605
3	5	92	18.2	25	4	15.630	17.346
3	6	92	21.2	25	4	16.170	14.753
3	7	92	24.2	25	4	15.837	12.905
3	8	92	18.4	25	3	21.097	12.685
3	9	92	20.4	25	3	21.139	11.495
3	10	92	22.4	25	3	21.780	10.360
3	0.5	92	19	25	15	4.263	61.333
4	1	92	17	20	10	6.300	45.827
4	2	92	17.6	20	7	9.005	31.353
4	3	92	19.8	20	6	10.645	23.531
4	4	92	20	20	5	12.884	19.745
4	5	92	18.2	20	4	16.149	16.990
4	6	92	21.2	20	4	15.908	14.765
4	7	92	24.2	20	4	16.365	12.764
4	8	92	18.4	20	3	21.901	12.497
4	9	92	20.4	20	3	21.285	11.335
4	10	92	22.4	20	3	21.296	10.384
4	0.5	92	19	20	15	4.307	60.577
5	1	82	17	15	10	5.750	45.475
5	2	82	17.6	15	7	8.164	30.801
5	3	82	19.8	15	6	9.676	22.983
5	4	82	20	15	5	11.429	19.384
5	5	82	18.2	15	4	14.581	16.675
5	6	82	21.2	15	4	14.240	14.884
5	7	82	24.2	15	4	14.686	12.697
5	8	82	18.4	15	3	19.596	12.457
5	9	82	20.4	15	3	19.035	11.321
5	10	82	22.4	15	3	19.266	10.351
5	0.5	82	17	15	15	3.804	60.773
6	1	96	17	15	10	6.761	44.355
6	2	96	17.6	15	7	9.569	30.801
6	3	96	19.8	15	6	11.266	23.234
6	4	96	20	15	5	13.600	19.516
6	5	96	18.2	15	4	17.198	16.748
6	6	96	21.2	15	4	16.706	14.559
6	7	96	24.2	15	4	17.263	12.637
6	8	96	18.4	15	3	23.149	12.401
6	9	96	20.4	15	3	22.791	11.153
6	10	96	22.4	15	3	22.292	10.261
6	0.5	96	19	15	15	4.531	60.348
7	1	86.6	17	12	10	6.070	44.843
7	2	86.6	17.6	12	7	8.639	30.288
7	3	86.6	19.8	12	6	10.203	23.169
7	4	86.6	20	12	5	12.337	18.975
7	5	86.6	18.2	12	4	15.432	16.650
7	6	86.6	21.2	12	4	15.242	14.758
7	7	86.6	24.2	12	4	15.613	12.564
7	8	86.6	18.4	12	3	20.805	12.338
7	9	86.6	20.4	12	3	20.249	11.237

7	10	86.6	22.4	12	3	20.543	10.267
7	0.5	86.6	19	12	15	4.130	59.973
8	1	80	17	10	10	5.646	44.777
8	2	80	17.6	10	7	8.082	30.103
8	3	80	19.8	10	6	9.356	22.965
8	4	80	20	10	5	11.500	18.685
8	5	80	18.2	10	4	14.028	16.939
8	6	80	21.2	10	4	14.462	14.099
8	7	80	24.2	10	4	14.079	12.736
8	8	80	18.4	10	3	18.748	12.617
8	9	80	20.4	10	3	19.171	11.320
8	10	80	22.4	10	3	19.299	10.099
8	0.5	80	19	10	15	3.823	59.863
9	1	89	17	10	10	6.366	44.318
9	2	89	17.6	10	7	9.019	29.741
9	3	89	19.8	10	6	10.453	23.237
9	4	89	20	10	5	12.828	18.748
9	5	89	18.2	10	4	15.902	16.612
9	6	89	21.2	10	4	15.793	14.653
9	7	89	24.2	10	4	16.153	12.484
9	8	89	18.4	10	3	21.529	12.263
9	9	89	20.4	10	3	20.928	11.172
9	10	89	22.4	10	3	21.136	10.224
9	0.5	89	19	10	15	4.219	59.921
10	1	98	17	10	10	6.996	43.776
10	2	98	17.6	10	7	9.849	30.549
10	3	98	19.8	10	6	11.774	22.692
10	4	98	20	10	5	13.805	19.442
10	5	98	18.2	10	4	17.748	16.774
10	6	98	21.2	10	4	17.476	14.250
10	7	98	24.2	10	4	17.320	12.638
10	8	98	18.4	10	3	23.244	12.436
10	9	98	20.4	10	3	23.865	11.076
10	10	98	22.4	10	3	23.599	10.057
10	0.5	98	19	10	15	4.632	59.805
0.5	1	90.5	17	70	10	5.558	51.688
0.5	2	90.5	17.6	70	7	7.959	35.698
0.5	3	90.5	19.8	70	6	9.408	26.640
0.5	4	90.5	20	70	5	11.172	21.671
0.5	5	90.5	18.2	70	4	14.464	18.868
0.5	6	90.5	21.2	70	4	13.937	16.329
0.5	7	90.5	24.2	70	4	14.319	14.202
0.5	8	90.5	18.4	70	3	19.796	13.610
0.5	9	90.5	20.4	70	3	19.575	12.321
0.5	10	90.5	22.4	70	3	18.890	11.330
0.5	0.5	90.5	19	70	15	3.741	69.097
